# Genes Associated with Thoracic Aortic Aneurysm and Dissection: 2018 Update and Clinical Implications

**DOI:** 10.1055/s-0038-1639612

**Published:** 2018-07-27

**Authors:** Adam J. Brownstein, Valentyna Kostiuk, Bulat A. Ziganshin, Mohammad A. Zafar, Helena Kuivaniemi, Simon C. Body, Allen E. Bale, John A. Elefteriades

**Affiliations:** 1 Department of Surgery, Section of Cardiac Surgery, Aortic Institute at Yale-New Haven Hospital, Yale University School of Medicine, New Haven, Connecticut; 2 Department of Surgical Diseases # 2, Kazan State Medical University, Kazan, Russia; 3 Division of Molecular Biology and Human Genetics, Department of Biomedical Sciences, and Department of Psychiatry, Faculty of Medicine and Health Sciences, Stellenbosch University, Tygerberg, South Africa; 4 Department of Anesthesiology, Perioperative and Pain Medicine, Brigham and Women's Hospital, Harvard Medical School, Boston, Massachusetts; 5 Department of Genetics, Yale School of Medicine, New Haven, Connecticut

**Keywords:** genetics, thoracic aortic aneurysm, thoracic aortic dissection

## Abstract

Thoracic aortic aneurysms, with an estimated prevalence in the general population of 1%, are potentially lethal, via rupture or dissection. Over the prior two decades, there has been an exponential increase in our understanding of the genetics of thoracic aortic aneurysm and/or dissection (TAAD). To date, 30 genes have been shown to be associated with the development of TAAD and ∼30% of individuals with nonsyndromic familial TAAD have a pathogenic mutation in one of these genes. This review represents the authors' yearly update summarizing the genes associated with TAAD, including implications for the surgical treatment of TAAD. Molecular genetics will continue to revolutionize the approach to patients afflicted with this devastating disease, permitting the application of genetically personalized aortic care.

This review is the update to the 2017 paper “Genes Associated with Thoracic Aortic Aneurysm and Dissection” published in AORTA. 1 We have updated both Table 1 listing the genes known to predispose to thoracic aortic aneurysm or dissection (TAAD) and Fig. 1 , with the recommended sizes for surgical intervention for each specific mutation, based upon published findings in 2017.

**Fig. 1:**
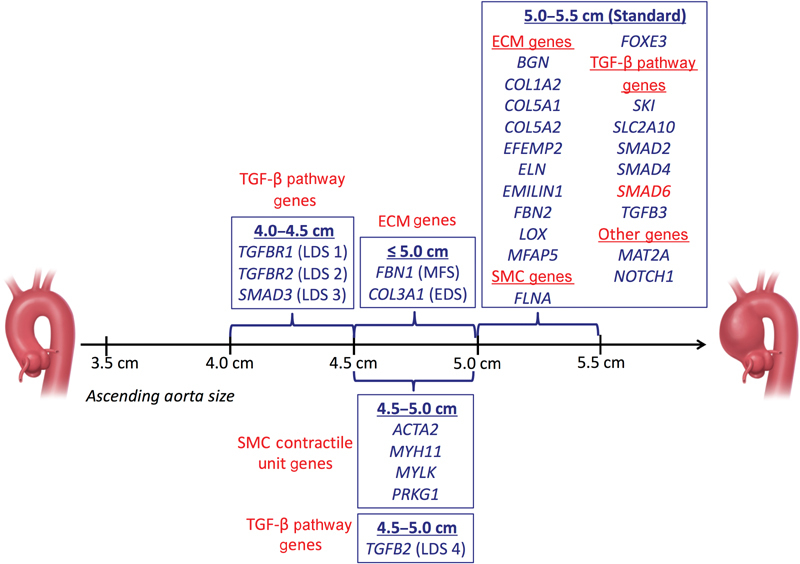
Ascending aorta dimensions for prophylactic surgical intervention. (Data derived from Table 1 and modified with permission from Brownstein et al. 1 ) Any gene newly reported during the past year to be associated with TAAD is highlighted in red. Abbreviations: ECM, extracellular matrix; SMC, smooth muscle cell; TAAD, thoracic aortic aneurysm and/or dissection; TGF, transforming growth factor.

**Table 1:** Genes associated with syndromic and nonsyndromic thoracic aortic aneurysm and/or dissection, associated vascular characteristics, and size criteria for elective surgical intervention (SMAD6 is the only gene that has been added to this table since publication of our 2017 AORTA review paper.)

Gene	Protein	Animal model leading to vascular phenotype?	Syndromic TAAD	Nonsyndromic FTAAD	Associated disease/syndrome	Associated clinical characteristics of the vasculature	Ascending Aorta Size (cm) for Surgical Intervention	Mode of inheritance	OMIM
*ACTA2*	Smooth muscle α-actin	Yes 10	+	+	AAT6 + multisystemic smooth muscle dysfunction + MYMY5	TAAD, early aortic dissection,* CAD, stroke (moyamoya disease), PDA, pulmonary artery dilation, BAV 11 12	4.5–5.0 a 13 14 15	AD	611788 613834 614042
*BGN*	Biglycan	Yes 16	+	−	Meester-Loeys syndrome	ARD, TAAD, pulmonary artery aneurysm, IA, arterial tortuosity 17	Standard	X-linked	300989
*COL1A2*	Collagen 1 α2 chain	No	+	−	EDS, arthrochalasia type (VIIb) + cardiac valvular type	Borderline aortic root enlargement 12 18	Standard	AD + AR	130060 225320
*COL3A1*	Collagen 3 α1 chain	Yes 19	+	−	EDS, vascular type (IV)	TAAD, early aortic dissection,* visceral arterial dissection, vessel fragility, IA 20 21 22	5.0 b 22	AD	130050
*COL5A1*	Collagen 5 α1 chain	No e	+	−	EDS, classic type 1	ARD, rupture/dissection of medium sized arteries 23 24 25	Standard	AD	130000
*COL5A2*	Collagen 5 α2 chain	Partially f	+	−	EDS, classic type 2	ARD	Standard	AD	130000
*EFEMP2*	Fibulin-4	Yes 26 27	+	−	Cutis laxa, AR type Ib	Ascending aortic aneurysms, other arterial aneurysms, arterial tortuosity and stenosis	Standard	AR	614437
*ELN*	Elastin	No	+	−	Cutis laxa, AD	ARD, ascending aortic aneurysm and dissection, BAV, IA possibly associated with SVAS 28 29 30	Standard	AD	123700 185500
*EMILIN1*	Elastin microfibril interfacer 1	No	+	−	Unidentified CTD	Ascending and descending aortic aneurysm 31	Standard	AD	Unassigned
*FBN1*	Fibrillin-1	Yes 32 33 34 35 36	+	+	Marfan syndrome	ARD, TAAD, AAA, other arterial aneurysms, pulmonary artery dilatation, arterial tortuosity 37	5.0 15 38	AD	154700
*FBN2*	Fibrillin-2	No	+	−	Contractual arachnodactyly	Rare ARD and aortic dissection, 39 BAV, PDA	Standard	AD	121050
*FLNA*	Filamin A	Yes 40 41	+	−	Periventricular nodular heterotopia	Aortic dilatation/aneurysms, peripheral arterial dilatation, 42 PDA, IA, 43 BAV	Standard	XLD	300049
*FOXE3*	Forkhead box 3	Yes 44	−	+	AAT11	TAAD (primarily Type A dissection) 44	Standard	AD	617349
*LOX*	Lysyl oxidase	Yes 45 46 47 48	−	+	AAT10	TAAD, AAA, hepatic artery aneurysm, BAV, CAD	Standard	AD	617168
*MAT2A*	Methionine adenosyltransferase II α	No g 49	−	+	FTAA	Thoracic aortic aneurysms, BAV 49	Standard	AD	Unassigned
*MFAP5*	Microfibril-associated glycoprotein 2	Partially h 50	−	+	AAT9	ARD, TAAD	Standard	AD	616166
*MYH11*	Smooth muscle myosin heavy chain	Partially i 51	−	+	AAT4	TAAD, early aortic dissection,* PDA, CAD, peripheral vascular occlusive disease, carotid IA	4.5–5.0 15 52	AD	132900
*MYLK*	Myosin light chain kinase	No j 53	−	+	AAT7	TAAD, early aortic dissections*	4.5–5.0 a 15 53	AD	613780
*NOTCH1*	NOTCH1	Partially k	−	+	AOVD1	BAV/TAAD 54 55	Standard	AD	109730
*PRKG1*	Type 1 cGMP-dependent protein kinase	No	−	+	AAT8	TAAD, early aortic dissection,* AAA, coronary artery aneurysm/dissection, aortic tortuosity, small vessel CVD	4.5–5.0 56	AD	615436
*SKI*	Sloan Kettering proto-oncoprotein	No l	+	−	Shprintzen–Goldberg syndrome	ARD, arterial tortuosity, pulmonary artery dilation, other (splenic) arterial aneurysms 57	Standard	AD	182212
*SLC2A10*	Glucose transporter 10	No m	+	−	Arterial tortuosity syndrome	ARD, 58 ascending aortic aneurysms, 58 other arterial aneurysms, arterial tortuosity, elongated arteries aortic/pulmonary artery stenosis	Standard	AR	208050
*SMAD2*	SMAD2	No	+	−	Unidentified CTD with arterial aneurysm/dissections	ARD, ascending aortic aneurysms, vertebral/carotid aneurysms and dissections, AAA 59 60	Standard	AD	Unassigned
*SMAD3*	SMAD3	Partially n 61	+	+	LDS type 3	ARD, TAAD, early aortic dissection,* AAA, arterial tortuosity, other arterial aneurysms/dissections, IA, BAV 62 63	4.0–4.2 15 38	AD	613795
*SMAD4*	SMAD4	Yes 64	+	−	JP/HHT syndrome	ARD, TAAD, AVMs, IA 65 66	Standard	AD	175050
*SMAD6*	SMAD6	No o	−	+	AOV2	BAV/TAA 6	Standard	AD	602931
*TGFB2*	TGF-β2	Yes 67	+	+	LDS type 4	ARD, TAAD, arterial tortuosity, other arterial aneurysms, BAV 67 68	4.5–5.0 c 69	AD	614816
*TGFB3*	TGF-β3	No p	+	−	LDS type 5	ARD, TAAD, AAA/dissection, other arterial aneurysms, IA/dissection 70	Standard	AD	615582
*TGFBR1*	TGF-β receptor type 1	Yes 71	+	+	LDS type 1 + AAT5	TAAD, early aortic dissection,* AAA, arterial tortuosity, other arterial aneurysms/dissection, IA, PDA, BAV 72	4.0–4.5 d, 15 38 73	AD	609192
*TGFBR2*	TGF-β receptor type 2	Yes 64 71	+	+	LDS type 2 + AAT3	TAAD, early aortic dissection,* AAA, arterial tortuosity, other arterial aneurysms/dissection, IA, PDA, BAV 72	4.0–4.5 d 15 38 73	AD	610168

Abbreviations: AAA, abdominal aortic aneurysm; AAT, aortic aneurysm, familial thoracic; AD, autosomal dominant; AOVD, aortic valve disease; AR, autosomal recessive; ARD, aortic root dilatation; AVM, arteriovenous malformation; BAV, bicuspid aortic valve; CAD, coronary artery disease; CTD, connective tissue disease; CVD, cerebrovascular disease; EDS, Ehlers–Danlos syndrome; FTAA, familial thoracic aortic aneurysm; FTAAD, familial thoracic aortic aneurysm and/or dissection; HHT, hereditary hemorrhagic telangiectasia; IA, intracranial aneurysm; JP, juvenile polyposis; LDS, Loeys-Dietz syndrome; MYMY, moyamoya disease; OMIM, Online Mendelian Inheritance in Man; PDA, patent ductus arteriosus; SVAS, supravalvular aortic stenosis; TGF, transforming growth factor; TAAD, thoracic aortic aneurysm and/or dissection; TGFBR, TGF-β receptor; XLD, X-linked dominant

It is important to note that since mutations in many of these genes are rare and have only recently been implicated in TAAD, there is a lack of adequate prospective clinical studies. Therefore, it is difficult to establish threshold diameters for intervention for TAAs, and each individual must be considered on a case by case basis, taking into account the rate of change in aneurysm size (> 0.5 cm per year is considered rapid), any family history of aortic dissection at diameters < 5.0 cm, and the presence of significant aortic regurgitation, which are all indications for early repair if present.

A “ + ” symbol in the syndromic TAAD column indicates that mutations in the gene have been found in patients with syndromic TAAD (same for the nonsyndromic TAAD column). A “-” symbol in the syndromic TAAD column indicates that mutations in the gene have not been found in patients with syndromic TAAD (same for the nonsyndromic TAAD column).

A reference is provided for each of the associated vascular characteristics not reported in the OMIM entry for that gene.

Standard = surgical intervention at 5.0 to 5.5 cm.

Early aortic dissection* = dissection at aortic diameters < 5.0 cm.

a Individuals with MYLK and ACTA2 mutations have been shown to have aortic dissections at a diameter of 4.0 cm. 13 53

b There are no data to set threshold diameters for the surgical intervention for EDS type IV. 38 The Canadian guidelines recommend surgery for aortic root sizes of 4.0 to 5.0 cm and ascending aorta sizes of 4.2 to 5.0 cm, though these patients are at high risk of surgical complications due to poor-quality vascular tissue. 74

c There are limited data concerning the timing of surgical intervention for LDS type 4. However, there has been a case of a type A aortic dissection at an aortic diameter < 5.0 cm 69 hence, the recommended threshold range of 4.5 to 5.0 cm.

d Current US guidelines recommend prophylactic surgery for LDS types 1 and 2 at ascending aortic diameters of 4.0 to 4.2 cm. 15 38 However, the European guidelines state that more clinical data are required. 22 Patients with TGFBR2 mutations have similar outcomes to patients with FBN1 mutations once their disease is diagnosed, 75 and the clinical course of LDS 1 and 2 does not appear to be as severe as originally reported. 73 76 77 Therefore, medically treated adult patients with LDS 1 or 2 may not require prophylactic surgery at ascending aortic diameters of 4.0 to 4.2 cm. 11 Individuals with TGFBR2 mutations are more likely to have aortic dissections at diameters < 5.0 cm than those with TGFBR1 mutations. 73 77 A more nuanced approach proposed by Jondeau et al utilizing the presence of TGFBR2 mutations (versus TGFBR1 mutations), the co-occurrence of severe systemic features (arterial tortuosity, hypertelorism, wide scarring), female gender, low body surface area, and a family history of dissection or rapid aortic root enlargement, which are all risk factors for aortic dissection, may be beneficial for LDS 1 and 2 patients to avoid unnecessary surgery at small aortic diameters. 73 Therefore, in LDS 1 or 2 individuals without the above features, Jondeau et al maintain that 4.5 cm may be an appropriate threshold, but females with TGFBR2 mutations and severe systemic features may benefit from surgery at 4.0 cm. 73

e Wenstrup et al found that mice heterozygous for an inactivating mutation in Col5a1 exhibit decreased aortic compliance and tensile strength relative to wild-type mice. 78

f Park et al recently demonstrated that Col5a2 haploinsufficiency increased the incidence and severity of AAA and led to aortic arch ruptures and dissections in an angiotensin II-induced aneurysm mouse model. 79 In an earlier paper, Park et al illustrated that mice heterozygous for a null allele in Col5a2 exhibited increased aortic compliance and reduced tensile strength compared with wild-type mice. 80

g Guo et al found that knockdown of mat2aa in zebrafish led to defective aortic arch development. 49

h Combs et al demonstrated that Mfap2 and Mfap5 double knockout (Mfap2 ^−/−^ ;Mfap5 ^−/−^ ) mice exhibit age-dependent aortic dilation, though this is not the case with Mfap5 single knockout mice.

i While Kuang et al reported that a mouse knock-in model (Myh11 ^R247C/R247C^ ) does not lead to a severe vascular phenotype under normal conditions, 81 Bellini et al demonstrated that induced hypertension in this mouse model led to intramural delaminations (separation of aortic wall layers without dissection) or premature deaths (due to aortic dissection based on necroscopy according to unpublished data by Bellini et al) in over 20% of the R247C mice, accompanied by focal accumulation of glycosaminoglycans within the aortic wall (a typical histological feature of TAAD).

j Wang et al demonstrated that SMC-specific knockdown of Mylk in mice led to histopathological changes (increased pools of proteoglycans) and altered gene expression consistent with medial degeneration of the aorta, though no aneurysm formation was observed.

k Koenig et el recently found that Notch1 haploinsufficiency exacerbates the aneurysmal aortic root dilation in a mouse model of Marfan syndrome and that Notch1 heterozygous mice exhibited aortic root dilation, abnormal smooth muscle cell morphology, and reduced elastic laminae. 82

l Doyle et al found that knockdown of paralogs of mammalian SKI in zebrafish led to craniofacial and cardiac anomalies, including failure of cardiac looping and malformations of the outflow tract. 57 Berk et al showed that mice lacking Ski exhibit craniofacial, skeletal muscle, and central nervous system abnormalities, which are all features of Shprintzen–Goldberg syndrome, but no evidence of aneurysm development was reported. 83

m Mice with homozygous missense mutations in Slc2a10 have not been shown to have the vascular abnormalities seen with arterial tortuosity syndrome, 84 though Cheng et al did demonstrate that such mice do exhibit abnormal elastogenesis within the aortic wall. 85

n Tan et al demonstrated that Smad3 knockout mice only developed aortic aneurysms with angiotensin II-induced vascular inflammation, though the knockout mice did have medial dissections evident on histological analysis of their aortas and exhibited aortic dilatation relative to wild-type mice prior to angiotensin II infusion. 61

o Galvin et al demonstrated that Madh6, which encodes Smad6, mutant mice exhibited defects in cardiac valve formation, outflow tract septation, vascular tone, and ossification but no aneurysm development was observed. 86

p Tgfb3 knockout mice die at birth from cleft palate 70 , but minor differences in the position and curvature of the aortic arches of these mice compared with wild-type mice have been described. 87

Thoracic aortic aneurysms, with an estimated prevalence in the general population of 1%, 2 are potentially lethal, via rupture or dissection. Although significant progress has been made in decreasing the mortality of type A and type B aortic dissections, particularly among individuals who are diagnosed and undergo surgical repair, 3 almost 50% of patients with a type A aortic dissection still die before hospital admission. 4 Therefore, it is critical for clinicians to identify those individuals at risk of TAAD and to perform clinical and genetic risk stratification so that appropriate and personalized management can be provided.

To date, 30 genes have been found to be associated with TAAD ( Table 1 and Fig. 1 ) and ∼30% of individuals with familial nonsyndromic TAAD (clinical manifestations restricted to the aorta) have a pathogenic variant in one or more of these genes. 5 Mutations in these genes lead to a spectrum of risk and severity of type A and B aortic dissections, 5 as well as different extra-aortic manifestations. Specific mutations in *ACTA2* are estimated to account for 12 to 21% of familial nonsyndromic TAAD, while mutations in syndromic genes ( *FBN1, TGFBR1, TGFBR2, SMAD3,* and *TGFB2* ) are estimated to account for an additional 14% of cases of familial nonsyndromic TAAD. 5 Other genes listed in Table 1 are estimated to contribute to 1 to 2% each or less of familial nonsyndromic TAAD. 5 Given that the majority of familial nonsyndromic TAAD cannot be explained by a mutation in one of the known genes associated with TAAD, it is likely that additional genes remain to be identified.

Several important genetic findings have been reported during the past year. Using exome sequencing of 441 patients with bicuspid aortic valve and thoracic aortic aneurysm, Gillis et al identified pathogenic mutations in *SMAD6* in 11 afflicted individuals, adding to the growing list of genes associated with TAAD. 6 Additionally, in an exome sequencing study of 27 patients with syndromic or familial TAAD (specifically focused on three pairs of first-degree relatives with the same pathogenic TAAD variant but differing phenotypic severity from three independent families), Landis et al found that variants within two genes, *ADCK4* and *COL15A1* , segregated with mild disease severity among thoracic aortic aneurysm patients, offering clues that may help explain the reduced penetrance and variable expression observed in those with TAAD. 7 Lastly, though not introducing a novel association, work by Franken et al on 290 Marfan syndrome (MFS) patients recently expanded our understanding of the genotype–phenotype relationships in TAAD—by demonstrating that among individuals with MFS, those with haploinsufficient mutations in *FBN1* have larger aortic root diameters that exhibit a more rapid dilation rate than those with dominant negative mutations. 8 Similarly, De Cario et al found that the presence of certain common polymorphisms in *TGFBR1* and *TGFBR2* was associated with reduced cardiovascular disease severity among patients with MFS. 9

These studies completed in 2017 illustrate the dynamic nature of the field of TAAD genetics. Through continued investigation and expanded access to genetic testing for affected patients and their family members, whole genome sequencing will undoubtedly continue to add new genes to the roster of causes for familial TAAD. Molecular genetics will continue to revolutionize the approach to patients afflicted with this devastating disease, permitting the application of genetically personalized aortic care. A major challenge in the field remains the lack of functional studies to prove the pathogenicity of identified variants.

We will continue to provide a yearly update and a revised summary table and revised intervention criterion table in AORTA at the end of each calendar year.

## References

[1] Brownstein A J Ziganshin B A Kuivaniemi H Body S C Bale A E Elefteriades J A Genes associated with thoracic aortic aneurysm and dissection: an update and clinical implications Aorta (Stamford) 2017 5 01 11 20 28868310 10.12945/j.aorta.2017.17.003 PMC5570562

[2] Verstraeten A Luyckx I Loeys B Aetiology and management of hereditary aortopathy Nat Rev Cardiol 2017 14 04 197 208 28102232 10.1038/nrcardio.2016.211

[3] Mody P S Wang Y Geirsson A Trends in aortic dissection hospitalizations, interventions, and outcomes among Medicare beneficiaries in the United States, 2000-2011 Circ Cardiovasc Qual Outcomes 2014 7 06 920 928 25336626 10.1161/CIRCOUTCOMES.114.001140 PMC4380171

[4] Howard D P Banerjee A Fairhead J F Perkins J Silver L E Rothwell P M; Oxford Vascular Study.Population-based study of incidence and outcome of acute aortic dissection and premorbid risk factor control: 10-year results from the Oxford Vascular Study Circulation 2013 127 20 2031 2037 23599348 10.1161/CIRCULATIONAHA.112.000483 PMC6016737

[5] Milewicz D M Regalado E Pagon R A Adam M P Ardinger H H Wallace S E Amemiya A Bean L JH Heritable Thoracic Aortic Disease OverviewIn:Seattle, WA GeneReviews(R) 1993 20301299

[6] Gillis E Kumar A A Luyckx I Candidate gene resequencing in a large bicuspid aortic valve-associated thoracic aortic aneurysm cohort: SMAD6 as an important contributor Front Physiol 2017 8 400 28659821 10.3389/fphys.2017.00400 PMC5469151

[7] Landis B J Schubert J A Lai D Exome sequencing identifies candidate genetic modifiers of syndromic and familial thoracic aortic aneurysm severity J Cardiovasc Transl Res 2017 10 04 423 432 28550590 10.1007/s12265-017-9753-1 PMC5702585

[8] Franken R Teixido-Tura G Brion M Relationship between fibrillin-1 genotype and severity of cardiovascular involvement in Marfan syndrome Heart 2017 103 22 1795 1799 28468757 10.1136/heartjnl-2016-310631

[9] De Cario R Sticchi E Lucarini L Role of TGFBR1 and TGFBR2 genetic variants in Marfan syndrome J Vasc Surg 2017 S0741-5214(17)31587-2. Article in Press 10.1016/j.jvs.2017.04.071 28847661

[10] Milewicz D M Prakash S K Ramirez F Therapeutics targeting drivers of thoracic aortic aneurysms and acute aortic dissections: insights from predisposing genes and mouse models Annu Rev Med 2017 68 51 67 28099082 10.1146/annurev-med-100415-022956 PMC5499376

[11] Milewicz D Hostetler E Wallace S Precision medical and surgical management for thoracic aortic aneurysms and acute aortic dissections based on the causative mutant gene J Cardiovasc Surg (Torino) 2016 57 02 172 177 26837258

[12] Bradley T J Bowdin S C Morel C F Pyeritz R E The expanding clinical spectrum of extracardiovascular and cardiovascular manifestations of heritable thoracic aortic aneurysm and dissection Can J Cardiol 2016 32 01 86 99 26724513 10.1016/j.cjca.2015.11.007

[13] Disabella E Grasso M Gambarin F I Risk of dissection in thoracic aneurysms associated with mutations of smooth muscle alpha-actin 2 (ACTA2) Heart 2011 97 04 321 326 21212136 10.1136/hrt.2010.204388

[14] Guo D C Pannu H Tran-Fadulu V Mutations in smooth muscle alpha-actin (ACTA2) lead to thoracic aortic aneurysms and dissections Nat Genet 2007 39 12 1488 1493 17994018 10.1038/ng.2007.6

[15] Andelfinger G Loeys B Dietz H A decade of discovery in the genetic understanding of thoracic aortic disease Can J Cardiol 2016 32 01 13 25 26724507 10.1016/j.cjca.2015.10.017

[16] Heegaard A M Corsi A Danielsen C C Biglycan deficiency causes spontaneous aortic dissection and rupture in mice Circulation 2007 115 21 2731 2738 17502576 10.1161/CIRCULATIONAHA.106.653980

[17] Meester J A Vandeweyer G Pintelon I Loss-of-function mutations in the X-linked biglycan gene cause a severe syndromic form of thoracic aortic aneurysms and dissections Genet Med 2017 19 04 386 395 27632686 10.1038/gim.2016.126 PMC5207316

[18] Schwarze U Hata R McKusick V A Rare autosomal recessive cardiac valvular form of Ehlers-Danlos syndrome results from mutations in the COL1A2 gene that activate the nonsense-mediated RNA decay pathway Am J Hum Genet 2004 74 05 917 930 15077201 10.1086/420794 PMC1181985

[19] Smith L B Hadoke P W Dyer E Haploinsufficiency of the murine Col3a1 locus causes aortic dissection: a novel model of the vascular type of Ehlers-Danlos syndrome Cardiovasc Res 2011 90 01 182 190 21071432 10.1093/cvr/cvq356 PMC3058731

[20] De Paepe A Malfait F The Ehlers-Danlos syndrome, a disorder with many faces Clin Genet 2012 82 01 1 11 22353005 10.1111/j.1399-0004.2012.01858.x

[21] Germain D P Ehlers-Danlos syndrome type IV Orphanet J Rare Dis 2007 2 32 17640391 10.1186/1750-1172-2-32 PMC1971255

[22] Erbel R Aboyans V Boileau C 2014 ESC guidelines on the diagnosis and treatment of aortic diseases: document covering acute and chronic aortic diseases of the thoracic and abdominal aorta of the adult Eur Heart J 2014 35 41 2873 2926 25173340 10.1093/eurheartj/ehu281

[23] Monroe G R Harakalova M van der Crabben S N Familial Ehlers-Danlos syndrome with lethal arterial events caused by a mutation in COL5A1 Am J Med Genet A 2015 167 06 1196 1203 25845371 10.1002/ajmg.a.36997

[24] Mehta S Dhar S U Birnbaum Y Common iliac artery aneurysm and spontaneous dissection with contralateral iatrogenic common iliac artery dissection in classic Ehlers-Danlos syndrome Int J Angiol 2012 21 03 167 170 23997563 10.1055/s-0032-1325118 PMC3578620

[25] Wenstrup R J Meyer R A Lyle J S Prevalence of aortic root dilation in the Ehlers-Danlos syndrome Genet Med 2002 4 03 112 117 12180144 10.1097/00125817-200205000-00003

[26] Huang J Davis E C Chapman S L Fibulin-4 deficiency results in ascending aortic aneurysms: a potential link between abnormal smooth muscle cell phenotype and aneurysm progression Circ Res 2010 106 03 583 592 20019329 10.1161/CIRCRESAHA.109.207852 PMC2826613

[27] Igoucheva O Alexeev V Halabi C M Fibulin-4 E57K knock-in mice recapitulate cutaneous, vascular and skeletal defects of recessive Cutis Laxa 1B with both elastic fiber and collagen fibril abnormalities J Biol Chem 2015 290 35 21443 21459 26178373 10.1074/jbc.M115.640425 PMC4571872

[28] Jelsig A M Urban Z Hucthagowder V Nissen H Ousager L B Novel ELN mutation in a family with supravalvular aortic stenosis and intracranial aneurysm Eur J Med Genet 2017 60 02 110 113 27866049 10.1016/j.ejmg.2016.11.004 PMC5843366

[29] Callewaert B Renard M Hucthagowder V New insights into the pathogenesis of autosomal-dominant cutis laxa with report of five ELN mutations Hum Mutat 2011 32 04 445 455 21309044 10.1002/humu.21462 PMC3383654

[30] Szabo Z Crepeau M W Mitchell A L Aortic aneurysmal disease and cutis laxa caused by defects in the elastin gene J Med Genet 2006 43 03 255 258 16085695 10.1136/jmg.2005.034157 PMC2563239

[31] Capuano A Bucciotti F Farwell K D Diagnostic exome sequencing identifies a novel gene, EMILIN1, associated with autosomal-dominant hereditary connective tissue disease Hum Mutat 2016 37 01 84 97 26462740 10.1002/humu.22920 PMC4738430

[32] Pereira L Andrikopoulos K Tian J Targeting of the gene encoding fibrillin-1 recapitulates the vascular aspect of Marfan syndrome Nat Genet 1997 17 02 218 222 9326947 10.1038/ng1097-218

[33] Pereira L Lee S Y Gayraud B Pathogenetic sequence for aneurysm revealed in mice underexpressing fibrillin-1 Proc Natl Acad Sci U S A 1999 96 07 3819 3823 10097121 10.1073/pnas.96.7.3819 PMC22378

[34] Judge D P Biery N J Keene D R Evidence for a critical contribution of haploinsufficiency in the complex pathogenesis of Marfan syndrome J Clin Invest 2004 114 02 172 181 15254584 10.1172/JCI20641 PMC449744

[35] Habashi J P Judge D P Holm T M Losartan, an AT1 antagonist, prevents aortic aneurysm in a mouse model of Marfan syndrome Science 2006 312(5770):117 121 16601194 10.1126/science.1124287 PMC1482474

[36] Lima B L Santos E J Fernandes G R A new mouse model for Marfan syndrome presents phenotypic variability associated with the genetic background and overall levels of Fbn1 expression PLoS One 2010 5 11 e14136 21152435 10.1371/journal.pone.0014136 PMC2994728

[37] Morris S A Orbach D B Geva T Singh M N Gauvreau K Lacro R V Increased vertebral artery tortuosity index is associated with adverse outcomes in children and young adults with connective tissue disorders Circulation 2011 124 04 388 396 21730308 10.1161/CIRCULATIONAHA.110.990549

[38] Hiratzka L F Bakris G L Beckman J A 2010 ACCF/AHA/AATS/ACR/ASA/SCA/SCAI/SIR/STS/SVM Guidelines for the diagnosis and management of patients with thoracic aortic disease. A Report of the American College of Cardiology Foundation/American Heart Association Task Force on Practice Guidelines, American Association for Thoracic Surgery, American College of Radiology, American Stroke Association, Society of Cardiovascular Anesthesiologists, Society for Cardiovascular Angiography and Interventions, Society of Interventional Radiology, Society of Thoracic Surgeons, and Society for Vascular Medicine J Am Coll Cardiol 2010 55 14 e27 e129 20359588 10.1016/j.jacc.2010.02.015

[39] Takeda N Morita H Fujita D Congenital contractual arachnodactyly complicated with aortic dilatation and dissection: case report and review of literature Am J Med Genet A 2015 167A 10 2382 2387 25975422 10.1002/ajmg.a.37162

[40] Retailleau K Arhatte M Demolombe S Smooth muscle filamin A is a major determinant of conduit artery structure and function at the adult stage Pflugers Arch 2016 468 07 1151 1160 27023351 10.1007/s00424-016-1813-x

[41] Feng Y Chen M H Moskowitz I P Filamin A (FLNA) is required for cell-cell contact in vascular development and cardiac morphogenesis Proc Natl Acad Sci U S A 2006 103 52 19836 19841 17172441 10.1073/pnas.0609628104 PMC1702530

[42] Reinstein E Frentz S Morgan T Vascular and connective tissue anomalies associated with X-linked periventricular heterotopia due to mutations in filamin A Eur J Hum Genet 2013 21 05 494 502 23032111 10.1038/ejhg.2012.209 PMC3641385

[43] Lange M Kasper B Bohring A 47 patients with FLNA associated periventricular nodular heterotopia Orphanet J Rare Dis 2015 10 134 26471271 10.1186/s13023-015-0331-9 PMC4608144

[44] Kuang S Q Medina-Martinez O Guo D C FOXE3 mutations predispose to thoracic aortic aneurysms and dissections J Clin Invest 2016 126 03 948 961 26854927 10.1172/JCI83778 PMC4767350

[45] Lee V S Halabi C M Hoffman E P Loss of function mutation in LOX causes thoracic aortic aneurysm and dissection in humans Proc Natl Acad Sci U S A 2016 113 31 8759 8764 27432961 10.1073/pnas.1601442113 PMC4978273

[46] Hornstra I K Birge S Starcher B Bailey A J Mecham R P Shapiro S D Lysyl oxidase is required for vascular and diaphragmatic development in mice J Biol Chem 2003 278 16 14387 14393 12473682 10.1074/jbc.M210144200

[47] Mäki J M Räsänen J Tikkanen H Inactivation of the lysyl oxidase gene Lox leads to aortic aneurysms, cardiovascular dysfunction, and perinatal death in mice Circulation 2002 106 19 2503 2509 12417550 10.1161/01.cir.0000038109.84500.1e

[48] Ren W Liu Y Wang X β-Aminopropionitrile monofumarate induces thoracic aortic dissection in C57BL/6 mice Sci Rep 2016 6 28149 27329825 10.1038/srep28149 PMC4916438

[49] Guo D C Gong L Regalado E S MAT2A mutations predispose individuals to thoracic aortic aneurysms Am J Hum Genet 2015 96 01 170 177 25557781 10.1016/j.ajhg.2014.11.015 PMC4289682

[50] Combs M D Knutsen R H Broekelmann T J Microfibril-associated glycoprotein 2 (MAGP2) loss of function has pleiotropic effects in vivo J Biol Chem 2013 288 40 28869 28880 23963447 10.1074/jbc.M113.497727 PMC3789982

[51] Bellini C Wang S Milewicz D M Humphrey J D Myh11(R247C/R247C) mutations increase thoracic aorta vulnerability to intramural damage despite a general biomechanical adaptivity J Biomech 2015 48 01 113 121 25433566 10.1016/j.jbiomech.2014.10.031 PMC4283495

[52] Pannu H Tran-Fadulu V Papke C L MYH11 mutations result in a distinct vascular pathology driven by insulin-like growth factor 1 and angiotensin II Hum Mol Genet 2007 16 20 2453 2462 17666408 10.1093/hmg/ddm201 PMC2905218

[53] Wang L Guo D C Cao J Mutations in myosin light chain kinase cause familial aortic dissections Am J Hum Genet 2010 87 05 701 707 21055718 10.1016/j.ajhg.2010.10.006 PMC2978973

[54] McKellar S H Tester D J Yagubyan M Majumdar R Ackerman M J Sundt T M III Novel NOTCH1 mutations in patients with bicuspid aortic valve disease and thoracic aortic aneurysms J Thorac Cardiovasc Surg 2007 134 02 290 296 17662764 10.1016/j.jtcvs.2007.02.041

[55] Proost D Vandeweyer G Meester J A Performant mutation identification using targeted next-generation sequencing of 14 thoracic aortic aneurysm genes Hum Mutat 2015 36 08 808 814 25907466 10.1002/humu.22802

[56] Guo D C Regalado E Casteel D E Recurrent gain-of-function mutation in PRKG1 causes thoracic aortic aneurysms and acute aortic dissections Am J Hum Genet 2013 93 02 398 404 23910461 10.1016/j.ajhg.2013.06.019 PMC3738837

[57] Doyle A J Doyle J J Bessling S L Mutations in the TGF-β repressor SKI cause Shprintzen-Goldberg syndrome with aortic aneurysm Nat Genet 2012 44 11 1249 1254 23023332 10.1038/ng.2421 PMC3545695

[58] Callewaert B L Willaert A Kerstjens-Frederikse W S Arterial tortuosity syndrome: clinical and molecular findings in 12 newly identified families Hum Mutat 2008 29 01 150 158 17935213 10.1002/humu.20623

[59] Micha D Guo D C Hilhorst-Hofstee Y SMAD2 mutations are associated with arterial aneurysms and dissections Hum Mutat 2015 36 12 1145 1149 26247899 10.1002/humu.22854

[60] Zhang W Zeng Q Xu Y Exome sequencing identified a novel SMAD2 mutation in a Chinese family with early onset aortic aneurysms Clin Chim Acta 2017 468 211 214 28283438 10.1016/j.cca.2017.03.007

[61] Tan C K Tan E H Luo B SMAD3 deficiency promotes inflammatory aortic aneurysms in angiotensin II-infused mice via activation of iNOS J Am Heart Assoc 2013 2 03 e000269 23782924 10.1161/JAHA.113.000269 PMC3698794

[62] van der Linde D van de Laar I M Bertoli-Avella A M Aggressive cardiovascular phenotype of aneurysms-osteoarthritis syndrome caused by pathogenic SMAD3 variants J Am Coll Cardiol 2012 60 05 397 403 22633655 10.1016/j.jacc.2011.12.052

[63] van de Laar I M van der Linde D Oei E H Phenotypic spectrum of the SMAD3-related aneurysms-osteoarthritis syndrome J Med Genet 2012 49 01 47 57 22167769 10.1136/jmedgenet-2011-100382

[64] Zhang P Hou S Chen J Smad4 deficiency in smooth muscle cells initiates the formation of aortic aneurysm Circ Res 2016 118 03 388 399 26699655 10.1161/CIRCRESAHA.115.308040

[65] Heald B Rigelsky C Moran R Prevalence of thoracic aortopathy in patients with juvenile polyposis syndrome-hereditary hemorrhagic telangiectasia due to SMAD4 Am J Med Genet A 2015 167A 08 1758 1762 25931195 10.1002/ajmg.a.37093

[66] Wain K E Ellingson M S McDonald J Appreciating the broad clinical features of SMAD4 mutation carriers: a multicenter chart review Genet Med 2014 16 08 588 593 24525918 10.1038/gim.2014.5 PMC4125531

[67] Lindsay M E Schepers D Bolar N A Loss-of-function mutations in TGFB2 cause a syndromic presentation of thoracic aortic aneurysm Nat Genet 2012 44 08 922 927 22772368 10.1038/ng.2349 PMC3616632

[68] Boileau C Guo D C Hanna N TGFB2 mutations cause familial thoracic aortic aneurysms and dissections associated with mild systemic features of Marfan syndrome Nat Genet 2012 44 08 916 921 22772371 10.1038/ng.2348 PMC4033668

[69] Renard M Callewaert B Malfait F Thoracic aortic-aneurysm and dissection in association with significant mitral valve disease caused by mutations in TGFB2 Int J Cardiol 2013 165 03 584 587 23102774 10.1016/j.ijcard.2012.09.029

[70] Bertoli-Avella A M Gillis E Morisaki H Mutations in a TGF-β ligand, TGFB3, cause syndromic aortic aneurysms and dissections J Am Coll Cardiol 2015 65 13 1324 1336 25835445 10.1016/j.jacc.2015.01.040 PMC4380321

[71] Gallo E M Loch D C Habashi J P Angiotensin II-dependent TGF-β signaling contributes to Loeys-Dietz syndrome vascular pathogenesis J Clin Invest 2014 124 01 448 460 24355923 10.1172/JCI69666 PMC3871227

[72] MacCarrick G Black J H III Bowdin S Loeys-Dietz syndrome: a primer for diagnosis and management Genet Med 2014 16 08 576 587 24577266 10.1038/gim.2014.11 PMC4131122

[73] Jondeau G Ropers J Regalado E International Registry of Patients Carrying TGFBR1 or TGFBR2 mutations: results of the MAC (Montalcino Aortic Consortium) Circ Cardiovasc Genet 2016 9 06 548 558 27879313 10.1161/CIRCGENETICS.116.001485 PMC5177493

[74] Boodhwani M Andelfinger G Leipsic J Canadian Cardiovascular Society position statement on the management of thoracic aortic disease Can J Cardiol 2014 30 06 577 589 24882528 10.1016/j.cjca.2014.02.018

[75] Attias D Stheneur C Roy C Comparison of clinical presentations and outcomes between patients with TGFBR2 and FBN1 mutations in Marfan syndrome and related disorders Circulation 2009 120 25 2541 2549 19996017 10.1161/CIRCULATIONAHA.109.887042

[76] Teixidó-Tura G Franken R Galuppo V Heterogeneity of aortic disease severity in patients with Loeys-Dietz syndrome Heart 2016 102 08 626 632 26848186 10.1136/heartjnl-2015-308535

[77] Tran-Fadulu V Pannu H Kim D H Analysis of multigenerational families with thoracic aortic aneurysms and dissections due to TGFBR1 or TGFBR2 mutations J Med Genet 2009 46 09 607 613 19542084 10.1136/jmg.2008.062844

[78] Wenstrup R J Florer J B Davidson J M Murine model of the Ehlers-Danlos syndrome. col5a1 haploinsufficiency disrupts collagen fibril assembly at multiple stages J Biol Chem 2006 281 18 12888 12895 16492673 10.1074/jbc.M511528200

[79] Park A C Phan N Massoudi D Deficits in Col5a2 expression result in novel skin and adipose abnormalities and predisposition to aortic aneurysms and dissections Am J Pathol 2017 187 10 2300 2311 28734943 10.1016/j.ajpath.2017.06.006 PMC5809516

[80] Park A C Phillips C L Pfeiffer F M Homozygosity and heterozygosity for null col5a2 alleles produce embryonic lethality and a novel classic Ehlers-Danlos syndrome-related phenotype Am J Pathol 2015 185 07 2000 2011 25987251 10.1016/j.ajpath.2015.03.022 PMC4483463

[81] Kuang S Q Kwartler C S Byanova K L Rare, nonsynonymous variant in the smooth muscle-specific isoform of myosin heavy chain, MYH11, R247C, alters force generation in the aorta and phenotype of smooth muscle cells Circ Res 2012 110 11 1411 1422 22511748 10.1161/CIRCRESAHA.111.261743 PMC3917690

[82] Koenig S N LaHaye S Feller J D Notch1 haploinsufficiency causes ascending aortic aneurysms in mice JCI Insight 2017 2 21 91353 29093270 10.1172/jci.insight.91353 PMC5752295

[83] Berk M Desai S Y Heyman H C Colmenares C Mice lacking the ski proto-oncogene have defects in neurulation, craniofacial, patterning, and skeletal muscle development Genes Dev 1997 11 16 2029 2039 9284043 10.1101/gad.11.16.2029 PMC316447

[84] Zoppi N Chiarelli N Cinquina V Ritelli M Colombi M GLUT10 deficiency leads to oxidative stress and non-canonical αvβ3 integrin-mediated TGFβ signalling associated with extracellular matrix disarray in arterial tortuosity syndrome skin fibroblasts Hum Mol Genet 2015 24 23 6769 6787 26376865 10.1093/hmg/ddv382 PMC4634379

[85] Cheng C H Kikuchi T Chen Y H Mutations in the SLC2A10 gene cause arterial abnormalities in mice Cardiovasc Res 2009 81 02 381 388 19028722 10.1093/cvr/cvn319

[86] Galvin K M Donovan M J Lynch C A A role for smad6 in development and homeostasis of the cardiovascular system Nat Genet 2000 24 02 171 174 10655064 10.1038/72835

[87] Azhar M Schultz J J Grupp I Transforming growth factor beta in cardiovascular development and function Cytokine Growth Factor Rev 2003 14 05 391 407 12948523 10.1016/s1359-6101(03)00044-3 PMC3855389

